# The moral determinants of health in South Africa: Diversity and inclusion in medicine

**DOI:** 10.4102/jcmsa.v2i1.80

**Published:** 2024-10-24

**Authors:** Brenda Z. Kubheka-Chauke

**Affiliations:** 1 Harvard Medical School, Center for Bioethics, Harvard University, Boston, United States of America; 2 Department of Clinical Risk and Ethics, Health IQ Consulting, Johannesburg, South Africa; 3 Programme within the School of Medicine, School of Medicine, Sefako Makgatho Health Sciences University, Tshwane, South Africa

**Keywords:** diversity, inclusion, belonging, equity, medical practitioners, discrimination, South Africa, justice

## Abstract

**Background:**

Diversity, inclusion, equity and access (DIEA) are fundamental principles deeply intertwined with South Africa’s historical narrative. Despite strides towards equality, the medical profession remains susceptible to challenges related to DIEA like the rest of society. Issues such as racial and gender bias, alongside covert discriminatory practices, persist within medical institutions. These concerns underscore the imperative for the medical community to prioritise diversity, inclusion and equitable access to opportunities throughout training and professional practice.

**Aim:**

This review aims to assess the current status of DIEA in South Africa, identify research and practice gaps and position South Africa within the global discourse on DIEA in medicine.

**Setting:**

Worldwide literature review was conducted to set a foundation for the analysis of the SA medical profession.

**Methods:**

A review of global literature was conducted to explore themes related to diversity, inclusion, access and equity in medicine.

**Results:**

Existing literature lacks sufficient focus on DIEA in the South African context, but highlights more United States-based discourse. There are limited empirical studies exploring systemic DIEA practices and its impact on access to opportunities and how it affects the well-being of underrepresented groups in medicine. These findings underscore the critical need for inclusive practices to drive both individuals and institutions to create a safe and just culture in medicine.

**Conclusion:**

Prioritising implementation and monitoring of inclusive policies and practices within the medical profession is imperative and should aim beyond numerical representation. Progress will be achieved through collaboration with bioethicists.

**Contribution:**

Providing insights and recommendations to advance DIEA in the South African field of medicine contributes to creating a more equitable and just medical profession.

## Introduction

Diversity has been touted as a business imperative because it facilitates innovation, better decision-making, retention of the less represented groups and other business or organisational benefits. Beyond the compelling business case, healthcare organisations should pursue diversity, inclusion, equity and access (DIEA) commitments because it is a moral imperative grounded on humanity. Humanity should have shared moral commitments based on respect, fairness, compassion and non-maleficence.^1^ Diversity, inclusion, equity and access enables the appreciation of similarities and differences between different groups of people, leading to a better understanding of the world and enabling organisations to solve complex problems. Differences such as life experiences and cultural backgrounds should be respected and grounded in compassion, cultural humility and seeking understanding. All humans have equal moral worth. Therefore, individuals should be respected because they have inherent dignity and share basic safety, esteem and self-actualisation needs. The same applies to equitable access to medical training and career advancement opportunities as the foundation for justice, which is fairness, in a world of inequality.

### Background

There is evidence that diversity of the healthcare team improves clinical outcomes.^2^ Therefore, DIEA should stem from leadership, team composition, clinical trials and service design, to name a few. Section 9 of the Constitution of South Africa guarantees:

[*T*]he right of every person not to be unfairly discriminated against, directly or indirectly, based on race, gender, sex, pregnancy, marital status, ethnic or social origins, colour, sexual orientation, age, disability, religion, conscience, belief, culture, language and birth.^3^

The composition of healthcare teams and institutional leaders must resemble the community’s demographic. Before 1940, South Africa (SA) did not provide opportunities for training in medicine for people who were not white, which means the care teams and leaders did not reflect the communities they served. The University of the Witwatersrand was the first medical school to admit black medical students in 1941.^4^

During apartheid, African, Indian and mixed race medical students experienced discriminatory training, which took place in segregated institutions. This segregation from their white counterparts constrained their professional careers. In the 1970s, mixed race and Indian interns earned 70%, and Africans interns earned 63% of white intern salaries.^5^ It is essential to acknowledge that post-apartheid SA has improved the diversity of medical practitioners and made their salaries and employee benefits, such as leave periods, just. Overt discrimination based on gender, race, ethnicity or other factors has become more elusive.^2^ However, the residue of apartheid remains, and today, it is reflected in the not fit-for-purpose old training hospitals previously designated for black Africans, such as George Mukhari Academic Hospital, the only institution, which was then a regional hospital and the second biggest in SA, linked to the former Medical University of South Africa (MEDUNSA) now Sefako Makgatho Health Sciences University, which was earmarked to train black health professionals. This differed from the University of the Witwatersrand medical school, linked to a specialised Rahima Moosa Mother and Child Hospital, Tara Psychiatric Hospital, Charlotte Maxeke Academic Hospital, Helen Joseph and Chris Hani Baragwanath Academic Hospitals. How many DIEA concepts are discussed during political and executive meetings is unknown. What is essential is ensuring that such discussions are sustained and go beyond examining race and gender statistics and focus attention on the intentional removal of structural factors perpetuating the disparities.^6^

Professor Ntusi, in his keynote address at the seminar hosted by the University of the Witwatersrand’s School of Public Health, highlighted that ‘the medical community has promoted structural racism through biased research and papers published in scientific journals’ and structural racism manifests as ‘perpetual inequity, deeply ingrained in social policy, legislation, law enforcement, the economic system, and the healthcare system’.^7^ Many individuals are instruments of oppression because they maintain and reproduce oppression in their daily activities because of the structural barriers maintained through bureaucratic processes normalising unjust practices.^8^ This article explores DIEA issues relevant to a medical career and its impact on workplace relationships, behaviour and decision-making. It is important to notice that there is a lack of South African literature on DIEA in healthcare.

## Discussion

Diverse, inclusive, equitable and accessible environments for healthcare training, research and care delivery are critical for advancing health equity.^9^ Institutional culture and climate influence the practice and values commended or tolerated, ultimately getting institutionalised. Healthcare organisations should recruit and retain professionals from diverse backgrounds and experiences.^9^ Individuals from previously marginalised groups should be encouraged to share their lived experiences and provide input to influence procedures and policies to be inclusive. While organisations must address representation intentionally, they must also be mindful of possible stigmatisation or poor perception of the worth of promoted leaders from marginalised or minority groups.^10^ South Africa has a two-tiered health system: private and public sectors, the latter serving most of the population. Diversity, inclusion, equity and access is essential in both sectors as they serve the same society.

### Navigating bias in medicine

Biases can be implicit, which is unconscious or conscious. Unconscious bias is ‘attitudes or stereotypes that unconsciously alter our perceptions or understanding of our experiences, thereby affecting behaviour, interactions, and decision-making’. Senior medical practitioners’ unconscious bias may impact trainees’ lived experiences, thus influencing their career decisions and the specialties they pursue.^2^ A study at a South African medical school highlighted the high prevalence of student mistreatment compared with international studies. Despite over 20 years of democracy in SA, there was evidence of high rates of racial and gender discrimination. Alarmingly, 83.4% of respondents experienced being ignored or excluded, 70% experienced offensive gestures, and 65.1% experienced verbal abuse.^11^

Bias is pervasive in society and the healthcare sector is not spared. A study involving a small group of black medical specialists in SA reported their experiences of feeling unwelcome and unrecognised. The respondents experienced everyday racism at work, including physical and psychological effects of tokenism, excessive pressure to excel and sexism.^12^ Some specialities are male dominated, which may have a negative impact on closing the gender gap. This gap can only be closed by recruiting and retaining others from minority or marginalised groups such as but not limited to lesbian, gay, bisexual, transgender, intersex, queer/questioning, asexual (LGBTQI+), women and people living with disabilities. Retaining these underrepresented groups requires effort to ensure leaders are equipped with skills for creating a conducive environment for underrepresented individuals to reach their full potential like their peers.

Other cases of sexism in medicine include a case involving several Japanese medical schools that were ordered by the court to pay damages to women for gender discrimination and emotional distress after being unfairly denied entry into medical schools. Shockingly, Tokyo Medical University admitted to rigging the admission applications for women to prevent them from entering medical training.^13^ One university interfered with the entrance exams, claiming that women ‘mature faster’ and male applicants need extra help, which qualifies as sexism. There were also allegations of raising exam scores of the children of former graduates in the hope that their parents would make donations to the school.^14,15^ Discrimination based on gender, social capital and other unjust measures is unfair and should be condemned.

Movements in the United States (US) such as #ILookLikeASurgeon highlight the struggles women doctors face because of gender biases and stereotypes. Other examples of systemic exclusions and inequities led to the #MeToo and #BlackLivesMatter movements.^10,13^ Biases are treacherous because they alter perceptions, behaviour, interactions and decision-making.^10,11,12^ Women doctors have been mistaken for nurses or addressed by their first names compared to their male counterparts, who are addressed formally with their titles. Women doctors also face unwanted sexual advances from peers, seniors and patients.^10^ Most people have biases influencing their perceptions and reactions.^16,17^ This includes a female scientist who is an associate dean at a medical school’s Office of Diversity who, according to the Implicit Association Test, is biased against women scientists.^17^ Many individuals hold biases about competency based on other irrelevant attributes, such as race, religion, sexual orientation, to name a few.^17^ Medical practitioners and leaders must use reasoning to counter automated behaviours and biased decision-making intentionally.^14^ Doing so will create better opportunities to act towards others with respect, compassion and fairness.

### Embracing diversity and inclusion in medicine

Diversity is defined as the presence of differences within a given setting. This may include gender, race, ethnicity, religion, nationality, sexual orientation, place of practice and practice type. Inclusion highlights who is considered a fellow and deserving respect and belonging in a group.^1^ Although medicine has become more diverse, noninclusive and hostile institutional culture still exist.^2^ Gender stereotypes about the role of women as home makers who must take care of children perpetuates biases and microaggressions faced in the workplace. Microaggressions are:

[*B*]rief and commonplace daily verbal or nonverbal behavioural, and environmental indignities whether intentional or unintentional that communicate hostile, derogatory or negative racial, ethnic, gender, sexual orientation, and religious slights and insults.^2^

These range from ‘women having high pitched voices or speaking softer’ to women not being thought of as leaders and this reflected on how male peers speak to them.^18,19^ Underrepresentation of women in medical institutions makes them vulnerable to sexual harassment, while medical institutions have an obligation to safeguard the well-being of all persons.^1^

Biases are innate tendencies to categorise everything, enabling quick interpretation and decision-making.^2^ These impact team dynamics and patient–practitioner relationships. An inclusive and diverse health professional workforce can significantly advance efforts to achieve health equity through improved access to culturally informed, person-centred and dignified care for vulnerable and underserved populations. The inclusion of minority groups such as LGBTQI+ should be purposeful to improve workplace hostility and address their lack of mentorship and scholarship recognition.^2^

Race plays a role in health disparities and the public is uninformed about the relationship between these variables. The awareness is also low among practitioners.^20,21^ A survey conducted among fellows of the American College of Surgeons cited that only 37% of respondents agreed that racial disparities existed in healthcare, 11% acknowledged that disparities existed in their hospital and only 4% acknowledged its existence in their practices.^22^ Most countries do not include race and racism in healthcare in their medical curricula, this includes South African medical schools despite the history of apartheid and prevalence of racism and other unjust discriminatory acts against particular groups in the society.

Inclusion is an ongoing intentional effort to ensure the full participation of diverse people in all aspects of an organisation, including decision-making.^23^ It is about making people feel they belong because they are seen, heard and respected despite their identities. Workplace discrimination impacts the job satisfaction and health of affected individuals, as cited in the study conducted by the National Health Services to assess the impact of increased discrimination and harassment.^24^ Similar conclusions were reached in a study conducted in a large medical academic centre, which established that perceived discrimination affects mental and physical well-being and the medical conditions observed were depression, anxiety, post-traumatic stress disorder, changes in sleeping patterns and blood pressure changes.^25^ Exclusion exists, and evidence speaks for itself. Neither ticking the boxes nor focusing on the inclusion business case will bring meaningful change. What is required are sustained, courageous conversations and actions about the impact of power imbalances, structural inequities and bias.^1^

### Tackling barriers to equity and access

Equity refers to an approach that ensures everyone can access the same opportunities. It recognises that advantages and barriers exist and that, as a result, everyone starts from different places.^23^ Generally, social factors, societal position and the underlying inequality influence access to opportunities.^18^ Justice concerns equal respect for all persons because of inherent dignity. Therefore, alternatives seeking to advance the interests of the most disadvantaged in society are just in pursuing fair equality of opportunity.^23,26^ Discriminatory barriers should be dismantled, and measures should be taken to correct unfair social practices grounded on biases or socioeconomic inequalities.

Greater health justice requires a multifaceted approach. Healthcare does not exist in isolation or a cocoon. The existing societal biases also transcend the training, promotions and recruitment of health professionals and research participants. An example of a widely used clinical tool, pulse oximetry, was proven less accurate in black patients, where a study cited nearly three times the frequency of occult hypoxemia that was not detected by pulse oximetry in white patients. One can only imagine the implications of this finding during the coronavirus disease 2019 (COVID-19) pandemic and the possible impact on delayed clinical care and outcomes.^27^ This is just one example highlighting the value of diversity among researchers and practitioners to raise some salient but missed factors. Diversity and access to opportunities not only go beyond discussing racial and gender disparities in healthcare and the effects of implicit bias but also through the deliberate inclusion of expert speakers and teachers from diverse backgrounds.^22^

Healthcare is undergoing fast-paced digital transformation and the adoption of automation. There is optimism about the benefits of artificial intelligence (AI) and concerns about the lack of transparency and algorithmic computations that may perpetuate biases and health disparities if not addressed.^28^ The challenge highlights the benefits of elevating DIEA in healthcare to ensure that the voices and concerns of health experts and marginalised and vulnerable populations are elevated to influence the design and deployment of these new technology tools.

A recently published study in the US concluded that a more excellent representation of black primary care physicians in the workforce was associated with better population health measures for black individuals.^29^ Inclusive care requires practitioners to put their understanding of equity into practice and is grounded on the premise that patients have different characteristics, needs and desires. Equitable access to inclusive care requires raising awareness of bias, communicating with inclusive language and embedding diversity concepts into training and care delivery.^30^ Fair distribution of moral determinants of health and access to opportunities for professionals are matters of justice.^31^

### The role of bioethicists in diversity, inclusion, equity and access

Bioethicists have a significant role in addressing issues related to DIEA in healthcare, particularly issues related to racism and other forms of discrimination. They can ensure that medical teaching involves engaging students in dialogue about racism and how to overcome it. Bioethicists can shift cultural competence in training, which tends to perpetuate stereotypes, to cultural humility, with the understanding of how experiences and socialisation influence people’s perceptions and interactions. Bioethicists can engage in outreach activities beyond the boundaries of the health system to facilitate conversations about such complex issues and mediate where required.^16^ The lack of research on the impact of DIEA in SA calls for bioethicists and other social scientists to collect primary data that will inform practice and facilitate inclusive policy development processes.^16^

## Conclusion

Promoting and implementing DIEA policies will advance health equity provided the objectives are standardised and measured numerically and qualitatively. Furthermore, including people from different groups who bring various experiences cultivates better problem-solving because of diverse perspectives and voices in healthcare management, care delivery, research and policy development. Caring about DIEA is insufficient; action is required to facilitate justice and belonging. A lack of respect for persons undermines their inherent dignity and their well-being. Medical schools and healthcare institutions must intentionally create awareness and commit resources to dismantle structural factors undermining DIEA. Fair and inclusive workplaces have better job satisfaction, well-being and staff retention, positively affecting patient care and experience. While DIEA is good for business, other organisations should pursue it as an end, for it is just and grounded on respect, equality and reciprocity. Empirical data specific to SA can assist in assessing the current culture and status of DIEA in the medical career. The insights can be a foundation for planning interventions and monitoring progress.
